# Multisystem Thromboembolic Shower in the Intensive Care Unit: Acute Limb Ischemia and Mesenteric Embolism

**DOI:** 10.1016/j.jaccas.2026.109328

**Published:** 2026-07-15

**Authors:** Wesley James Cox, Caleb Brown, Lay Pandya

**Affiliations:** a Department of Internal Medicine, Iredell Health System, Statesville, North Carolina, USA; b Department of Internal Medicine, Carilion Clinic, Roanoke, Virginia, USA

**Keywords:** aspirin, atherosclerosis, atrial fibrillation, peripheral vascular disease, thrombosis, thrombus

## Abstract

**Background:**

Acute limb ischemia is a catastrophic vascular emergency that can easily be obscured by the physiological noise of septic shock and mechanical ventilation within the intensive care unit.

**Case Summary:**

A 75-year-old Caucasian woman presented to the emergency department and was subsequently admitted to the intensive care unit with acute hypoxic respiratory failure and severe septic shock secondary to a primary lobar bacterial pneumonia. She was obtunded, tachycardic, and hypotensive upon arrival. She experienced a transient 4-hour paroxysm of new-onset atrial fibrillation. Targeted physical examination revealed a profoundly cold right lower extremity with absent peripheral pulses. Emergent computed tomography angiography revealed a complex, multisystem macrovascular catastrophe: an acute superior mesenteric artery occlusion alongside a 15-mm right common iliac artery thrombus causing near-subtotal occlusion. Prompt systemic anticoagulation and surgical thrombectomy successfully preserved bowel viability and avoided a major lower limb amputation.

**Discussion:**

Widespread multibed embolic showers require high clinical suspicion when brief tachyarrhythmias interact with the hypercoagulable cascade of systemic sepsis.

**Take-Home Message:**

Systematic peripheral vascular assessments are vital in complex sepsis presentations to prevent devastating diagnostic delays.

## Visual Summary

**Figure:**
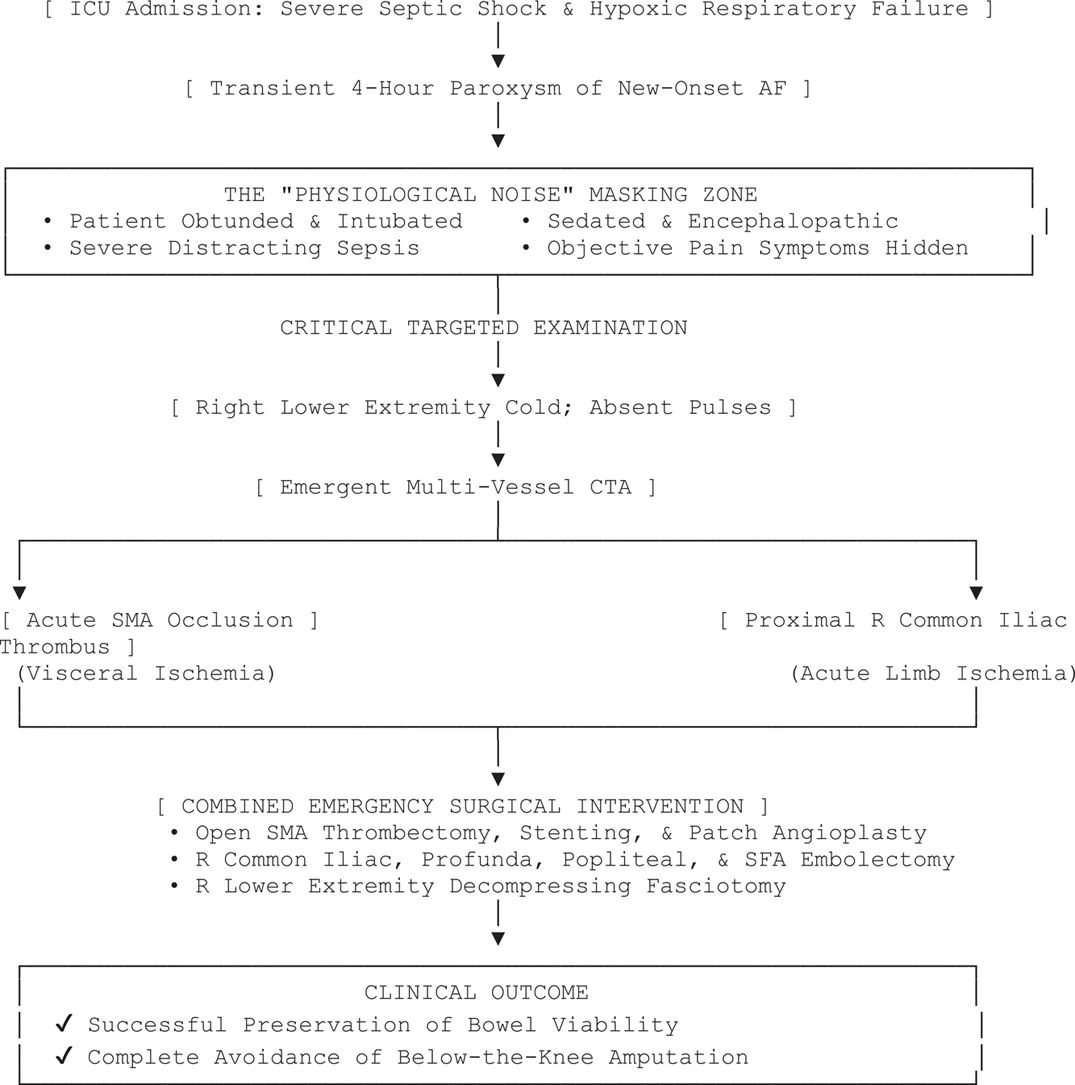

**Visual Summary:**
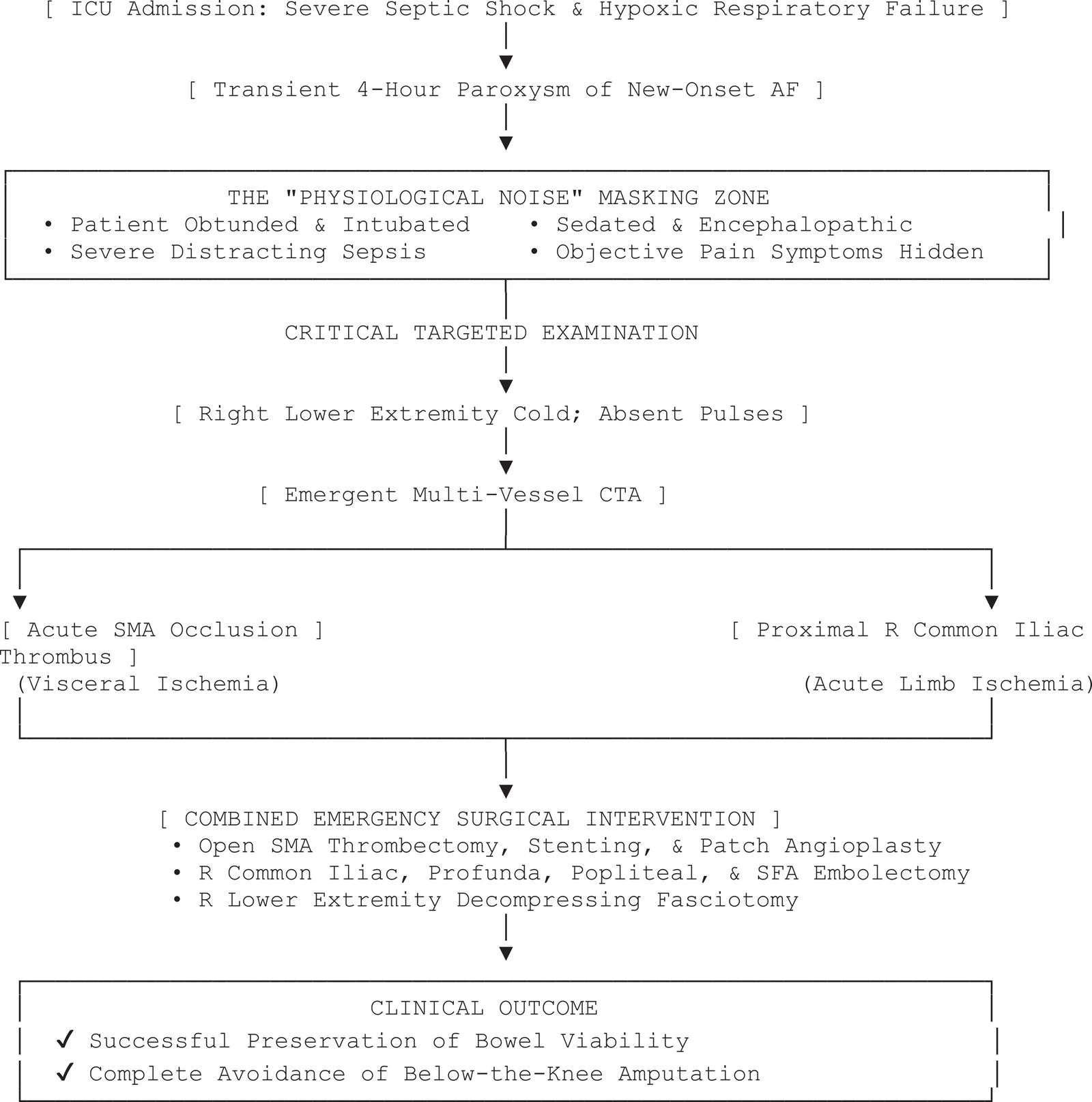
Chronological Flow and Teaching Points of Multibed Thromboembolism Mapping of the clinical course from the initial 4-hour paroxysm of atrial fibrillation to the recognition of lower limb pulselessness and synchronous superior mesenteric artery occlusion, outlining diagnostic workflows to circumvent masking by ICU sedation and septic shock parameters. AF = atrial fibrillation; CTA = computed tomography angiography; ICU = intensive care unit; R = right; SFA = superficial femoral artery; SMA = superior mesenteric artery.

## Medical History

A 75-year-old Caucasian woman presented to the emergency department and was subsequently admitted to the intensive care unit (ICU) with acute hypoxic respiratory failure and severe septic shock secondary to a primary lobar bacterial pneumonia. Upon arrival to the ICU, the patient was obtunded, tachycardic, and hypotensive. Her past medical history was notable for severe chronic obstructive pulmonary disease, essential hypertension, and hyperlipidemia. Social history detailed a significant 20 pack-year tobacco smoking history.

**Take-Home Message**

• Systematic peripheral vascular assessments are vital in complex sepsis presentations to prevent devastating diagnostic delays.

Initial emergency laboratory metrics revealed severe metabolic lactic acidosis with an initial lactate of 9.0 mmol/L, a marked leukocytosis of 12.7 × 10^3^/μL, and an acute kidney injury (creatinine 1.49 mg/dL from a baseline of 0.90 mg/dL).^1^ Platelet counts and baseline coagulation profiles were entirely within normal limits.

The patient's outpatient home medications included an albuterol inhaler, lisinopril, and atorvastatin, with no prior history of outpatient or home anticoagulation. During initial stabilization, an electrocardiogram captured a new-onset episode of paroxysmal atrial fibrillation (AF) with rapid ventricular response peaking at 122 beats/min, which persisted for approximately 4 hours before spontaneously reverting to sinus tachycardia. Given her rapidly declining mental status, severe encephalopathy, and a Glasgow coma scale score of 10, the patient was intubated, sedated, and initiated on standard critical care algorithms for fluid-refractory septic shock.

## Investigations

Upon arrival at the medical ICU, a targeted physical examination was performed, which critically noted that the patient's right lower extremity was profoundly cold to the touch with completely absent palpable and Dopplerable dorsalis pedis and posterior tibial pulses. Given the combination of her earlier transient 4-hour tachyarrhythmia, acute abdominal tenderness before intubation, and acute lower limb poikilothermia, an emergent computed tomography angiography (CTA) of the chest, abdomen, pelvis, and lower extremities was performed.

**Figure 1:**
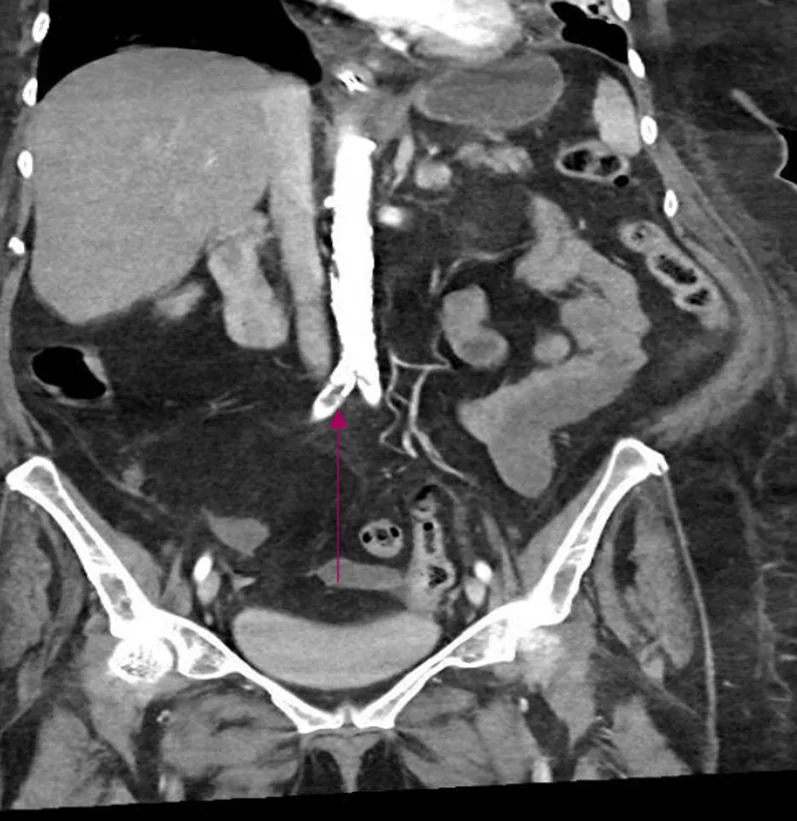
Acute Superior Mesenteric Artery Occlusion Coronal computed tomography angiography displaying the distal abdominal aorta splitting into the common iliac arteries. A large, prominent filling defect is tracking through the proximal right common iliac artery (arrow), causing subtotal luminal obstruction and a critical drop in downstream peripheral perfusion.

**Figure 2:**
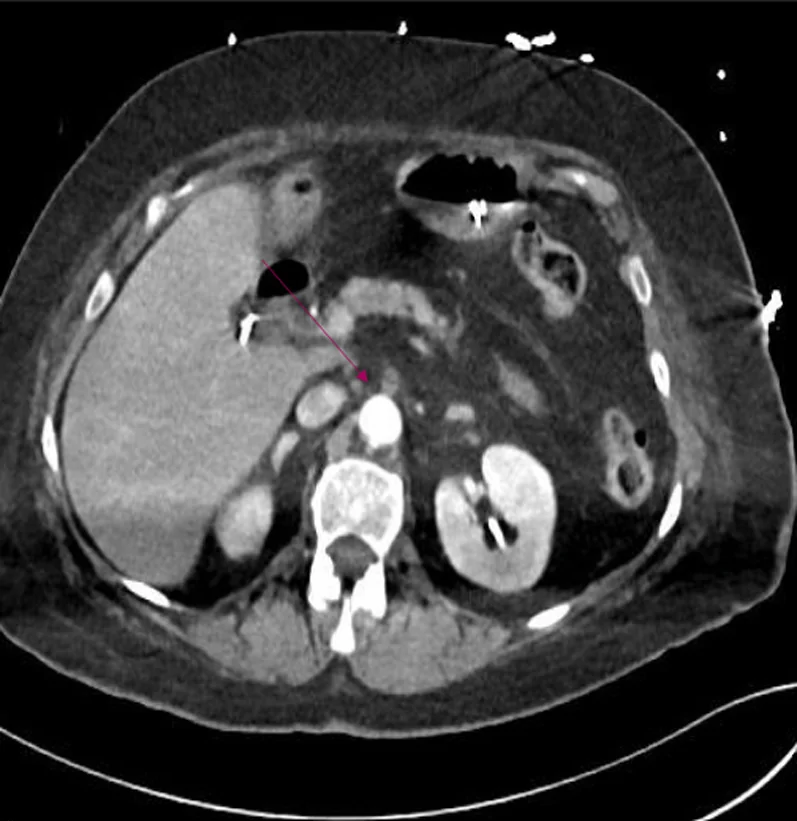
Terminal Aorta Bifurcation and Lower Extremity Runoff Axial computed tomography angiography slice obtained at the level of the lower lumbar spine showing the terminal abdominal aorta bifurcation. A distinct, noncalcified, low-attenuation filling defect is visible within the lumen of the right common iliac artery (arrow), representing acute embolic entrapment.

The CTA revealed a complex, multisystem macrovascular catastrophe: a major acute filling defect within the proximal branch of the superior mesenteric artery (SMA) resulting in acute occlusion with surrounding mesenteric fat stranding, alongside a concurrent acute thrombus within the proximal right common iliac artery causing near-subtotal luminal occlusion (Figures 1 and 2).^2^^,^^3^ The thrombus propagated distally to result in multilevel segmental occlusions of the right common femoral, profunda femoris, and popliteal arteries down through the trifurcation vessels. Proximal screening of the chest confirmed a normal thoracic aorta contour, ruling out an acute aortic dissection or floating thoracic atheroma.

To delineate the source of this widespread embolic event, an urgent transthoracic echocardiogram was performed. The echocardiogram revealed a preserved left ventricular ejection fraction of 55%, no significant valvular disease, and no patent foramen ovale or atrial septal defect; however, it demonstrated left atrial stasis with prominent spontaneous echo contrast (“smoke”) within the left atrium, confirming a high-risk intracardiac milieu secondary to her paroxysmal atrial fibrillation. A detailed breakdown of the daily diagnostic findings and clinical milestones is summarized in Table 1.

**Table 1:** Clinical Course and Management Timeline

Date	Clinical Status and Interventions	Diagnostic and Laboratory Findings
February 1	• Emergent ICU admission for septic shock. • Started on continuous weight-based heparin infusion. • Transient 4-h paroxysm of new-onset atrial fibrillation. • Emergent multivessel surgical embolectomy (R profunda, popliteal, SFA) + SMA thrombectomy and stenting. • Right lower extremity fasciotomy performed due to compartment syndrome risk.	• Initial profound lactic acidosis (lactate: 9.0 mmol/L). • Multivessel CTA confirmation of acute common iliac and proximal SMA occlusions. • Postintervention loss of Doppler signals; foot mottling noted.
February 2	• Initiated nutritional support via tube feeds. • Scheduled for a right lower extremity guillotine amputation due to suspected irreversible limb ischemia.	• Serum lactate normalized. • CT head returned negative for acute intracranial pathology. • Hypercoagulability panel drawn (protein C, factor V, APLS).
February 3	• Guillotine amputation deferred after clinical improvement. • Aggressive fluid resuscitation initiated for acute rhabdomyolysis. • Successfully weaned off all vasopressor support.	• Return of palpable right lower extremity Doppler pulses. • TTE: Normal EF, no PFO/ASD, no structural defects. • Blood cultures positive for MRSA and *H* *influenzae* bacteremia.
February 4	• Antiarrhythmic therapy transitioned from IV amiodarone to oral Diltiazem for heart rate control.	• Chest x-ray: Bibasilar opacities and small pleural effusions. • Creatine kinase levels stabilized and began trending downward.
February 5	• Left and right lower extremity pulses intact and stable; below-the-knee amputation successfully avoided. • Surgical consultation obtained to evaluate potential bowel ischemia secondary to ileus.	• Repeat CT abdomen/pelvis: Confirmed patent SMA stents; no signs of frank bowel necrosis. • Lactic acid remained completely normal (1.1 mmol/L).

APLS = antiphospholipid syndrome; ASD = atrial septal defect; CT = computed tomography; CTA = computed tomography angiography; EF = ejection fraction; ICU = intensive care unit; IV = intravenous; MRSA = methicillin-resistant *Staphylococcus aureus*; PFO = patent foramen ovale; SMA = superior mesenteric artery; TTE = transthoracic echocardiogram.

## Differential Diagnosis

The differential diagnosis for simultaneous, multiterritory macrovascular occlusion in a critically ill patient centers on cardioembolic versus macrovascular thrombotic etiologies. Given the documented 4-hour paroxysm of new-onset AF and left atrial smoke on echocardiography, a central cardioembolic shower was the leading diagnosis. Competing macrovascular conditions included an acute type B aortic dissection propagating into the visceral and iliac origins, which was definitively excluded by the normal thoracic aorta configuration on CTA.^4^

Primary in situ arterial thrombosis secondary to an underlying inherited or acquired hypercoagulable state—such as antiphospholipid syndrome, protein C or S deficiency, antithrombin III deficiency, or factor V Leiden mutation—was highly prioritized. Additionally, acute atherosclerotic plaque rupture with downstream atheroembolism was evaluated but was deemed less likely given the simultaneous involvement of 2 separate, noncontiguous major vascular beds.

### Management/Clinical Course

The patient was immediately started on a continuous weight-based intravenous heparin infusion. Given the acute Rutherford class IIb status of the right lower extremity and the high risk of transmural bowel necrosis, she was taken for emergency surgical intervention on February 1.

Vascular surgery teams successfully performed a right common iliac, profunda, popliteal, and superficial femoral artery surgical embolectomy, combined with an open SMA thrombectomy and stenting/patch angioplasty. A right lower extremity decompressive fasciotomy was executed concurrently to manage reperfusion injury and compartment syndrome risk.

On February 2, the patient experienced a transient postintervention loss of distal Doppler signals and foot mottling. She was provisionally scheduled for a right lower extremity guillotine amputation because of suspected irreversible limb ischemia. However, aggressive fluid resuscitation and optimization of systemic perfusion parameters led to a remarkable clinical turnaround, allowing the guillotine amputation to be successfully deferred on February 3. Her systemic shock resolved, and she was successfully weaned off all vasopressor support.

Antiarrhythmic therapy was transitioned from intravenous amiodarone to oral diltiazem for stable heart rate control on February 4. Serum creatine kinase levels, which peaked near 5,000 U/L due to acute rhabdomyolysis, stabilized and trended downward. Blood cultures identified the primary lobar pneumonia source, turning positive for methicillin-resistant *Staphylococcus aureus* and *Haemophilus influenzae* bacteremia, which were treated with tailored intravenous vancomycin and cefepime. On February 5, a repeat computed tomography scan of the abdomen confirmed patent SMA stents with no signs of frank bowel necrosis, and her lactic acid remained completely normal at 1.1 mmol/L.

## Discussion

Synchronous macrovascular thromboembolic events affecting both visceral and peripheral limb beds represent an extreme clinical crisis with an incredibly narrow therapeutic window. Skeletal muscle tolerates warm ischemia for approximately 4 to 6 hours before irreversible tissue necrosis ensues, while intestinal mucosa undergoes transmural infarction within a similar timeframe.

This case highlights how the typical symptoms of vascular occlusion—such as severe abdominal pain and lower extremity claudication—can be completely masked by encephalopathy, deep sedation, and the distracting physiological noise of septic shock and respiratory failure within the ICU environment. The development of new-onset paroxysmal AF, even if brief, can act as a catastrophic embolic engine when interacting with the hypercoagulable cascade of underlying systemic sepsis.

To rule out an underlying prothrombotic state, a comprehensive hypercoagulability panel (including factor V Leiden, prothrombin mutation, protein C/S functional activity, antithrombin III levels, and antiphospholipid antibodies) was completed 6 weeks postdischarge and returned entirely normal. This definitive outpatient work-up confirmed that the macroembolic shower was acutely driven by critical illness hypercoagulability and paroxysmal atrial fibrillation rather than an intrinsic hematological defect.

### Follow-up

Following a prolonged ICU course, the patient's neurological status returned to her baseline. Her right lower extremity fasciotomy wounds healed appropriately with healthy granulation tissue, and distal peripheral pulses remained intact and stable, allowing a below-the-knee amputation to be successfully avoided. She was transitioned from intravenous heparin to long-term oral anticoagulation therapy for secondary embolic prevention and was safely discharged to a rehabilitation facility.

## Conclusions

Early detection of acute limb ischemia and synchronous visceral ischemia requires an exceptionally high index of clinical suspicion in the ICU environment. Critical care teams must look past the distracting parameters of severe respiratory failure or septic illness and perform systematic physical examinations—including serial peripheral pulse evaluations and regular Doppler assessments—whenever a new tachyarrhythmia or unexplained metabolic derangement arises.

## Funding Support and Author Disclosures

The authors have reported that they have no relationships relevant to the contents of this paper to disclose.
