# CORRECTION

**DOI:** 10.1016/j.jaccas.2026.110731

**Published:** 2026-10-21

Cox WJ, Brown C, Pandya L

**Multisystem Thromboembolic Shower in the Intensive Care Unit: Acute Limb Ischemia and Mesenteric Embolism**

*JACC Case Rep*. 2026;31:109328.

The authors apologize for the following errors.

The current online version has been corrected.

In the Case Summary section of the Abstract, the final sentence (beginning “Prompt systemic anticoagulation…”) should be:

The patient subsequently developed bowel ischemia and died in the ICU. Prompt anticoagulation prevented progression to major lower-extremity limb loss, but bowel viability was not ultimately preserved.

In the Management Clinical Course section, 4th paragraph, the final sentence (beginning “On February 5…”) should be:

On February 5, repeat computed tomography of the abdomen demonstrated a patent superior mesenteric artery (SMA) stent; however, several small bowel loops appeared hypo enhancing, and bowel ischemia could not be entirely excluded. There were also multiple dilated, fluid-filled small bowel loops without a discrete transition point, consistent with ileus. Her lactate initially normalized on February 4 but subsequently demonstrated a progressive increase, reaching 2.5 mmol/L.

Immediately following the replacement sentence described in the above, the following 2 paragraphs have been added:

On February 6, the patient developed worsening fevers and hypoxic respiratory failure requiring mechanical ventilation with an FiO_2_ of 100%. She subsequently developed worsening metabolic and lactic acidosis and required maximal vasopressor support, raising significant concern for progressive bowel ischemia. Attempts at left subclavian central venous access were complicated by thrombosis involving the right internal jugular and left subclavian veins, as well as superficial thrombosis of the right radial artery, raising concern for disseminated intravascular coagulation. Given concern for heparin resistance additionally, anticoagulation was transitioned to argatroban.

The patient was evaluated by general surgery; however, given her profound hemodynamic instability and critical illness, she was not considered a surgical candidate. With continued clinical deterioration, she was ultimately transitioned to comfort-focused care.

In the Discussion section, the entire 3rd paragraph (beginning “To rule out an underlying…” through the sentence ending “…an intrinsic hematological defect”) should be removed. It should not be replaced with any new text.

The entirety of the Follow-up section—from the “Follow-up” header through the sentence that ends “…discharged to a rehabilitation facility” —should be removed. It should not be replaced with any new text.

Figure 1 and its legend should be replaced with the following:

**Figure 1:**
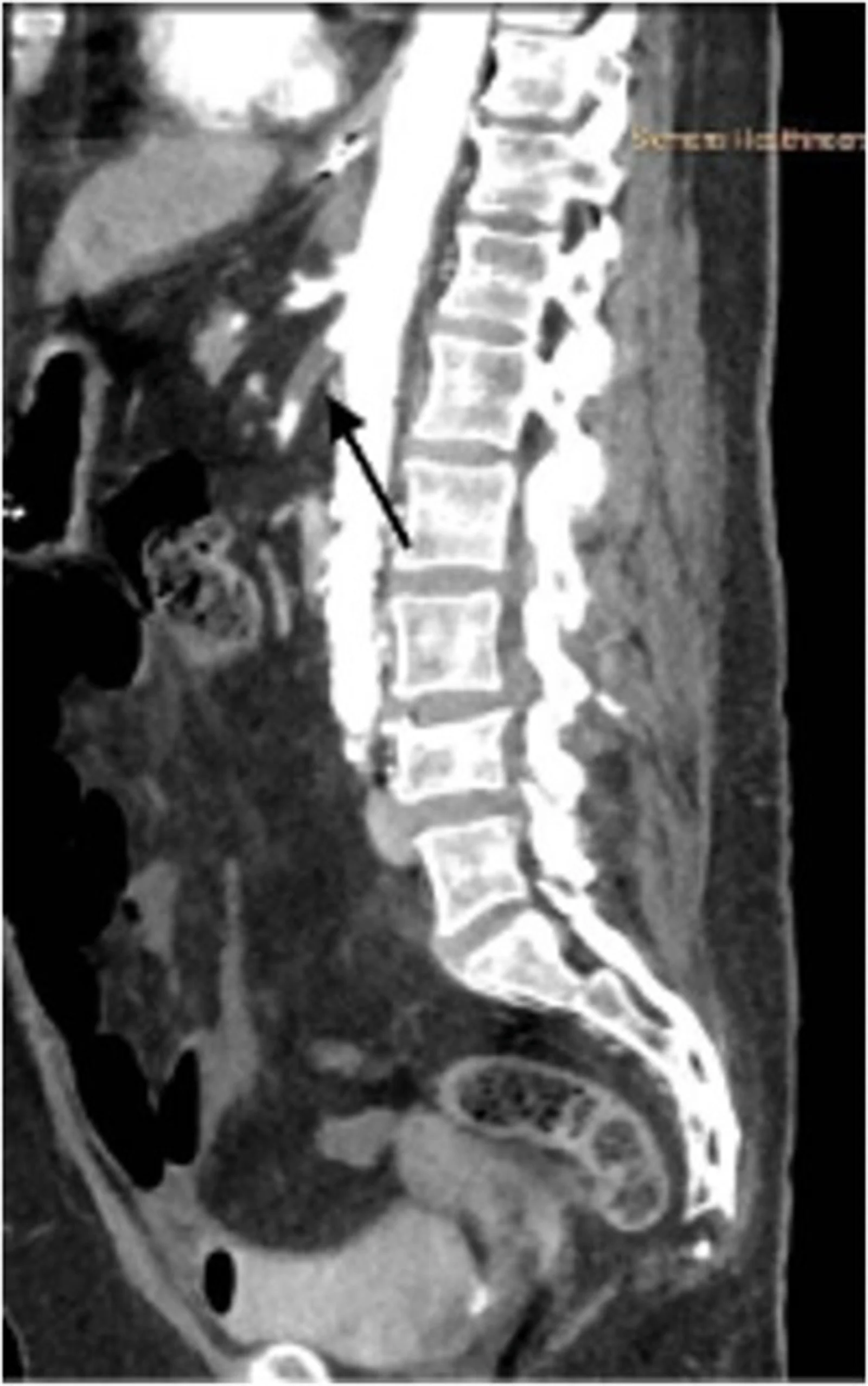
Acute Superior Mesenteric Artery Occlusion Sagittal computed tomography angiography demonstrating an intraluminal filling defect with occlusion of the proximal superior mesenteric artery (arrow), resulting in impaired distal mesenteric arterial perfusion.

Figure 2 and its legend should be replaced with the following:

**Figure 2:**
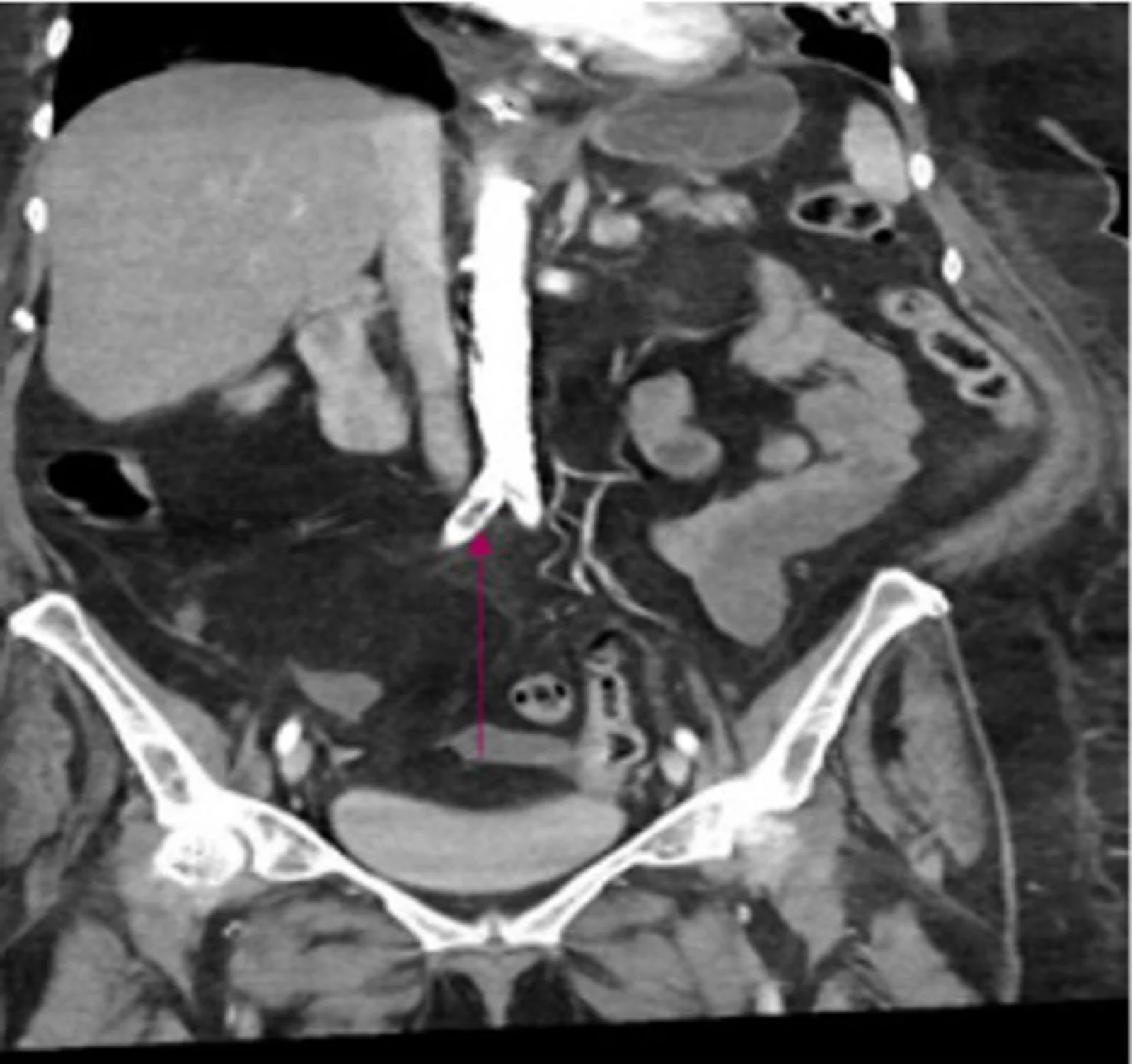
Terminal Aorta Bifurcation and Lower Extremity Runoff Coronal computed tomography angiography displaying the distal abdominal aorta splitting into the common iliac arteries. A large, prominent filling defect is tracked through the proximal right common iliac artery (arrow), causing subtotal luminal obstruction and a critical drop in downstream peripheral perfusion.

