# Supplementary material for: Multisystem Thromboembolic Shower in the Intensive Care Unit: Acute Limb Ischemia and Mesenteric Embolism
Source: JACC Case Rep. 2026 Jul 15;31(34):109328. doi: 10.1016/j.jaccas.2026.109328 (PMC13531209; doi:10.1016/j.jaccas.2026.109328)
Supplement: Supplementary file 1 [file mmc1.docx]

**Supplemental Figure 1:**

**Supplemental Figure 2:**

**Supplemental Figure Legend:** “Supplemental Figure 1 & 2: Sagittal CTA reconstructions of the thoracic aorta demonstrating a completely normal luminal diameter, regular wall contours, and total absence of an intimal dissection flap or mobile atheroma, ruling out a primary aortic catastrophe.”
